# Neurological Complications of COVID-19: Underlying Mechanisms and Management

**DOI:** 10.3390/ijms22084081

**Published:** 2021-04-15

**Authors:** Ghaydaa A. Shehata, Kevin C. Lord, Michaela C. Grudzinski, Mohamed Elsayed, Ramy Abdelnaby, Hatem A. Elshabrawy

**Affiliations:** 1 Department of Neurology and Psychiatry, Assiut University Hospitals, Assiut 71511, Egypt; ghaidaa.abozaid@med.aun.edu.eg; 2 Department of Physiology and Pharmacology, College of Osteopathic Medicine, Sam Houston State University, Conroe, TX 77304, USA; kcl043@shsu.edu; 3 College of Osteopathic Medicine, Sam Houston State University, Conroe, TX 77304, USA; mcg043@shsu.edu; 4 Department of Psychiatry and Psychotherapy III, University of Ulm, Leimgrubenweg 12-14, 89075 Ulm, Germany; mohamed.elsayed@uni-ulm.de; 5 Department of Neurology, RWTH Aachen University, Pauwelsstraße 30, 52074 Aachen, Germany; rabdelnaby@ukaachen.de; 6 Department of Molecular and Cellular Biology, College of Osteopathic Medicine, Sam Houston State University, Conroe, TX 77304, USA

**Keywords:** SARS-CoV-2, COVID-19, encephalitis, encephalopathy, seizures, neurological, management, cerebrovascular, stroke, Guillain–Barré syndrome, headache, myalgia, dizziness

## Abstract

COVID-19 is a severe respiratory disease caused by the newly identified human coronavirus (HCoV) Severe Acute Respiratory Syndrome Coronavirus-2 (SARS-CoV-2). The virus was discovered in December 2019, and in March 2020, the disease was declared a global pandemic by the World Health Organization (WHO) due to a high number of cases. Although SARS-CoV-2 primarily affects the respiratory system, several studies have reported neurological complications in COVID-19 patients. Headache, dizziness, loss of taste and smell, encephalitis, encephalopathy, and cerebrovascular diseases are the most common neurological complications that are associated with COVID-19. In addition, seizures, neuromuscular junctions’ disorders, and Guillain–Barré syndrome were reported as complications of COVID-19, as well as neurodegenerative and demyelinating disorders. However, the management of these conditions remains a challenge. In this review, we discuss the prevalence, pathogenesis, and mechanisms of these neurological sequelae that are secondary to SARS-CoV-2 infection. We aim to update neurologists and healthcare workers on the possible neurological complications associated with COVID-19 and the management of these disease conditions.

## 1. Introduction

In December 2019, the novel coronavirus, Severe Acute Respiratory Syndrome Coronavirus-2 (SARS-CoV-2), was identified as the causative agent of the acute atypical cluster of pneumonia cases in the city of Wuhan, China [1]. In February 2020, the World Health Organization (WHO) named the disease COVID-19 [2]. Although initially identified by respiratory symptoms, there have been increasing reports describing the copresentation of nonspecific neurological symptoms, including headache, dizziness, fatigue, and myalgia, impacting greater than 80% of all hospitalized patients [3]. What had previously been described only by pulmonary symptoms is now recognized by multiple neurological complications. Current data correlate the acuity of COVID-19 and mortality in critical care patients to the severity of neurological diseases, including acute necrotizing encephalopathy, encephalitis, epilepsy/seizures, and ataxia, increasing the risk of brain damage [4]. Additionally, peripheral nervous system (PNS) complications have been reported, including hypogeusia, hyposmia, Guillain–Barré syndrome, and skeletal muscle injury [5].

In this review, we aim to provide updates to the most current neurological complications resulting from COVID-19 and the treatment guidelines for these conditions.

## 2. Search Strategy and Selection Criteria

We performed a systematic search on PubMed utilizing the search terms “Coronavirus and Neurological,” “SARS-COV-2 and Neurological,” and “COVID-19 and management strategies (neurological or stroke or encephalitis or encephalopathy or seizures)” published between January 2019 and February 2021, yielding 5378 articles. We further filtered for articles in English, yielding 5212. After duplications and articles not relevant to the purpose of this review, we evaluated over 750 publications resulting in the 241 we used to support our review.

## 3. Coronaviruses and Neurological Complications

The blood–brain and blood–cerebrospinal fluid (CSF) barriers are structured to prevent the invasion of the brain by pathogens and toxic molecules [6]; however, this is not totally impermeable. There are multiple mechanisms by which neurotropic viruses are able to traverse the blood–brain barrier (BBB), but the most common route is the hematogenous route, which starts by entering the bloodstream causing viremia [7,8]. Once in the blood, viruses are able to cross the BBB via transcytosis or the infection of endothelial cells [9,10], infected monocytes (“Trojan Horse” mechanism) [9], and paracellularly via disrupted tight junctions in the endothelial cells due to inflammation caused by the viremia [7,9]. Another route not dependent on viremia includes the coordination of dynein and kinesins proteins in the transport of the virus into the CNS using infected motor or sensory nerves [7]. Viruses can also enter the CNS through olfactory sensory neurons [7]. The latter is a more common route for respiratory coronaviruses [10].

To date, seven CoVs have been associated with diseases in humans, which include HCoV-OC43, HCoV-229E, HCoV-NL63, HCoV-HKU1, Middle East Respiratory Syndrome-CoV (MERS-CoV), Severe Acute Respiratory Syndrome Coronavirus (SARS-CoV), and most recently SARS-CoV-2 [11,12,13,14]. Only SARS-CoV, MERS-CoV, and SARS-CoV-2 are recognized as causative agents of severe respiratory diseases, whereas all other human coronaviruses (HCoVs) typically present as mild diseases [15,16,17].

Not different from many other viruses [7,18], CoVs are known to cause neurological complications [19,20,21,22,23]. However, the neuroinvasive mechanisms have not been well understood. Murray et al. presented the first evidence of the association of HCoVs with neurological disease in multiple sclerosis (MS) patients [24,25], with later studies confirming HCoV-229E and HCoV-OC43 in patients diagnosed with Parkinson’s disease, Schizophrenia, Alzheimer’s disease, and meningoencephalitis [26]. A potential mechanism for CNS infection was suggested in a 2004 case study reporting the presence of HCoV-OC43 in nasopharyngeal and CSF samples of a child who was diagnosed with acute disseminated encephalomyelitis [19]. These findings were supported by St-Jean et al., who described the route of HCoV-OC43 infection of mice CNS through the olfactory bulb seven days after a nasal infection leading to acute encephalitis [20]. Additional murine studies confirmed the development of acute encephalitis in HCoV-OC43-infected BALB/c mice and HCoV-OC43-induced apoptosis in mice and rat neuronal cells [27]. These findings highlight the neurotropic characteristics of HCoVs and their ability to infect the CNS.

Soon after the emergence of SARS-CoV in 2002–2003, neurological complications were reported in SARS-CoV patients [28]. In addition to regular symptoms, such as fever, chills, productive cough, and diarrhea, patients developed neurological complications such as seizures, convulsions, and loss of consciousness during the course of the disease [29]. Tests later confirmed the presence of SARS-CoV in the CSF. With the new awareness of the pervasiveness of the disease, the examination of samples from patients who have died of SARS-CoV has revealed the presence of the SARS-CoV-N protein and viral RNA in several organs, including the stomach, small intestine, kidney, sweat glands, liver, and cerebrum [30]. The neurotropic property of SARS-CoV was further confirmed by a study in C57BL/6 mice, which showed that intranasal infection of mice eventually led to the infection of mice brain [31]. The previous findings indicate that SARS-CoV is capable of causing systemic infections, including CNS infections. In 2016, a study showed that some children who suffered acute encephalitis had a concurrent HCoV infection [32].

Similar to other HCoVs, SARS-CoV-2 has been associated with neurological complications, which are now recognized as initial symptoms in conjunction with the typical respiratory manifestations [33]. The most common neurological manifestations include headache, lethargy, unstable gait, ataxia, and seizures, in addition to PNS manifestations such as loss of taste and smell, vision impairment, nerve pain, and malaise [34]. The most serious developing neurological diseases include polyneuritis, Guillain–Barré syndrome (GBS), meningitis, encephalitis, and encephalopathy, in addition to cerebral hemorrhage and infarction [34]. Liotta et al., in a study of 509 COVID-19 patients, showed that 82% of these patients experienced neurological complications, which manifest early in 42% of patients and in 63% of patients at hospitalization [3]. Adjusting for age and severity of disease, younger patients and those presenting with severe COVID-19 are more likely to present with neurological manifestations, while older patients are more likely to develop a neurological disease (encephalopathies). These findings have been further supported by another study of 214 patients. In this study, 78 patients (36.4%) suffered from neurological consequences to COVID-19 [35]. These neurological complications manifest as CNS-related complications, such as dizziness, headache, impaired consciousness, acute cerebrovascular disease, ataxia, and seizure, or as PNS manifestations, such as loss of taste and smell, vision impairment, and nerve pain, as well as skeletal muscular injury. There was a higher incidence of neurological complications in patients with severe COVID-19 than in mild COVID-19 patients.

All the previous manifestations depend on the SARS-CoV-2 infection of host target cells; primarily unciliated bronchial epithelial cells and type II pneumocytes in the lung, after binding to cell surface receptors; angiotensin-converting enzyme 2 (ACE2), basigin (BSG; CD147), and neuropilin-1 (NRP-1) [36,37,38]. Cellular proteases such as TMPRSS2, furin, and cathepsins are required for priming the viral spike (S) protein, a process that is essential for viral entry after binding to host cell receptors [36]. Human brain single-nuclear RNA sequencing (RNA-seq) data suggest low or no expression of ACE2 on different human brain cells and its microvasculature [39]. However, higher expression of other SARS-CoV-2 receptors, such as BSG and NRP-1, was reported in many brain cell types [39]. Moreover, host cell proteases are also expressed at different levels in most brain cells [39]. The previous findings suggest that the brain may be susceptible to SARS-CoV-2 invasion and infection.

## 4. Mechanisms of SARS-CoV-2 Invasion of the CNS

Studies have reported the presence of SARS-CoV-2 in the CSF and postmortem brain tissue of COVID-19 patients with encephalitis [40,41,42,43,44,45,46,47,48]. However, there are contradictory findings that may indicate that the neurological complications are due to severe systemic inflammation and not the direct invasion of the brain [49,50,51,52,53,54,55,56].

**Figure 1:**
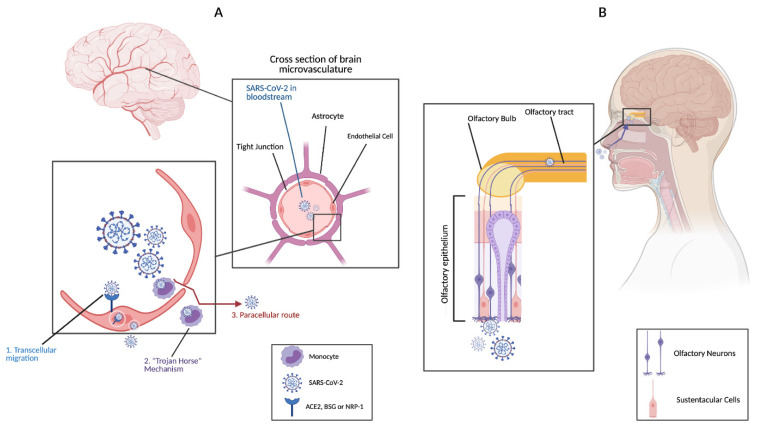
Mechanisms of SARS-CoV-2 invasion of the CNS. (**A**) Hematogenous route: Severe Acute Respiratory Syndrome Coronavirus-2 (SARS-CoV-2) invasion of CNS from the bloodstream is mediated by three mechanisms; 1. Transcellular migration which involves binding of the virus to its receptors; ACE2, basigin (BSG), or neuropilin-1 (NRP-1), on brain microvasculature endothelial cells then crossing endothelial cells via transcytosis, 2. Infecting immune cells which then carry the virus across the blood–brain barrier (BBB) endothelial cells into the CNS (Trojan Horse mechanism), and 3. Paracellular route by disrupting endothelial cells’ tight junctions. (**B**) SARS-CoV-2 infects olfactory epithelium and reaches the CNS via the olfactory neurons. This figure was created with BioRender.com.

It has been suggested that SARS-CoV-2 could invade the CNS via the same routes as other HCoVs [hematogenous route (Figure 1A) or by using retrograde or antegrade transport mechanisms from peripheral nerves to the CNS (Figure 1B)] [33,40,57,58,59,60,61].

One possible mechanism of the hematogenous route is binding to SARS-CoV-2 receptors on BBB endothelial cells, passing through endothelial cells by transcytosis, and finally reaching the brain (Figure 1A) [40,62]. The infection of endothelial cells does not involve any viral replication [33]. Because BSG and NRP1 are more highly expressed than ACE2 in the brain microvasculature, it is more likely that the SARS-CoV-2 would utilize these receptors to enter the CNS [39]. The other proposed mechanism involves infecting immune cells that express ACE2, such as monocytes, granulocytes, and lymphocytes, (“Trojan horse” mechanism) (Figure 1A) [63,64,65,66,67]. The infected immune cells may then carry SARS-CoV-2 to the CNS, where it can infect the brain [68]. SARS-CoV-2 viral RNA was detected in the lung macrophages of COVID-19 patients; however, viral replication in immune cells and immune infiltration of the brain need to be confirmed [69]. One additional mechanism is passing through disrupted endothelial cells’ tight junctions (paracellular route) (Figure 1A).

As mentioned earlier, SARS-CoV-2 may also reach the CNS via peripheral nerves, more specifically the olfactory sensory neurons (Figure 1B) [57,58]. The high expression of ACE2 and the priming protease, transmembrane serine protease 2 (TMPRSS2), in sustentacular cells, stem cells of the olfactory epithelium, and olfactory bulb may allow for retrograde or antegrade transport into the CNS [61,70,71,72,73,74].

## 5. Neurological Disorders and Their Management in COVID-19 Patients

### 5.1. Cerebrovascular Diseases

**Figure 2:**
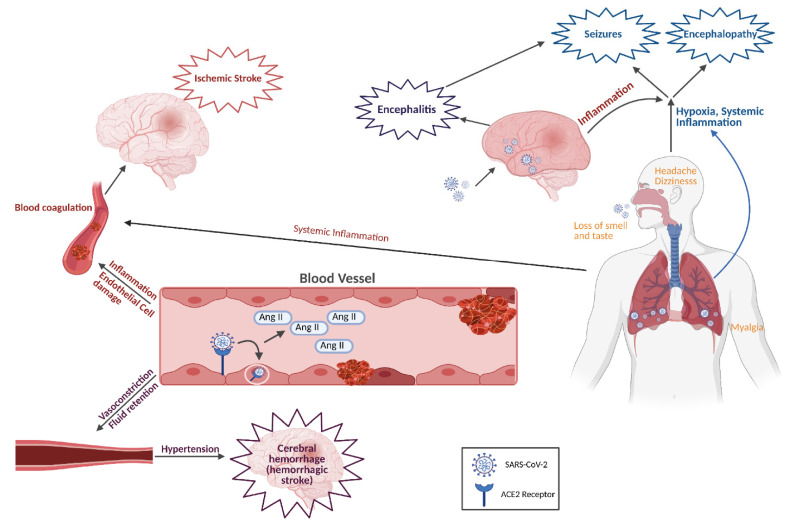
Mechanisms of COVID-19 neurological complications. Lung infection by SARS-CoV-2 results in severe inflammation, acute respiratory distress syndrome (ARDS), and hypoxia. This leads to hypoxia- and inflammation-induced encephalopathy and seizures. Brain damage due to viral replication may lead to encephalitis. Severe systemic inflammation could result in hypercoagulability which may eventually lead to stroke. Nonspecific symptoms due to nervous system affections include headache, dizziness, loss of taste and smell, and myalgia. Usage of ACE2 receptor; by SARS-CoV-2, to infect target cells, including endothelial cells, would deplete the receptor resulting in the accumulation of angiotensin II (AngII). High levels of AngII promote vasoconstriction, fluid retention, inflammation, and blood coagulation, which could result in ischemic or hemorrhagic stroke. This figure was created with BioRender.com.

Cerebrovascular complications have been documented in 5% of COVID-19 patients, with 60% of these events attributed to an acute ischemic stroke [35,75,76]. The increased risk of these events is believed to be due to a hyperinflammatory/hypercoagulable state, and altered endothelial cell function resulting from the SARS-CoV-2 infection (Figure 2) [77,78,79,80,81]. Several studies have reported a significant increase in neutrophil-to-lymphocyte ratio (NLR), C-reactive protein (CRP), and serum ferritin in COVID-19 patients with ischemic stroke, which could predict mortality in these patients [82,83,84,85,86,87]. Neutrophilia (increase in neutrophils) described in these patients results in the overproduction of neutrophil extracellular traps (NETs), which has been shown to increase thrombi formation [88,89,90]. Furthermore, hypercoagulability and the increase in thrombi formation in COVID-19 patients could be explained by impaired fibrinolysis, low levels of natural anticoagulants, and high levels of coagulation factors and antiphospholipid antibodies [91,92,93,94]. The formation of thrombi is further potentiated by SARS-CoV-2-mediated damage of the endothelium, which results in nitric oxide synthase (NOS) depletion and subsequent deficiency of NO [95]. NO deficiency increases the risk of stroke because NO is a potent vasodilator and an inhibitor of platelets and leukocytes adhesion to the endothelium [95].

Moreover, the internalization of ACE2, following the binding of SARS-CoV-2, leads to ACE2 depletion on the surface of endothelial cells, which may increase the incidence of ischemic stroke [96]. Data have shown a significant reduction in ACE2 expression on endothelial cells of SARS-CoV-2 patients [97]. Lack of ACE2 leaves angiotensin II (Ang II), a powerful vasoconstrictor, unregulated, thus increasing the risk of hypertension, blood coagulation, and ischemic stroke (Figure 2).

A case study has reported acute stroke-like symptoms and intracranial hypertension in a 75-year-old Australian man due to severe inflammatory response to COVID-19 [98]. The neurological involvement in this case was not discovered until Day 26 postinfection, which highlights the importance of clinical values, such as NLR, lymphocyte-to-CRP ratio (LCRPR), and lymphocyte-to-platelet ratio (LPR), as prognostic indicators of severe inflammation and possible neurological complications. Other studies have shown that COVID-19-induced severe inflammation and inflammatory infiltrates consisting of T cells, macrophages, and neutrophils contribute to the rupture of atheromatous plaques in patients with pre-existing atheromatous disease due to the production of proteolytic enzymes and endothelial cell disruption [99,100,101]. Although the use of protease inhibitors in these patients may be beneficial, they should be carefully used as they may promote SARS-CoV-2-induced hypercoagulation.

Following the occurrence of ischemic stroke, the production of proinflammatory mediators from activated immune cells and ischemic brain tissue could further promote brain injury [102,103]. Therefore, the suppression of inflammation in ischemic stroke could help ameliorate brain damage following ischemic stroke. However, further studies are needed to prove the therapeutic utility of this approach.

In addition to ischemic stroke, intracranial hemorrhage was observed in 0.5% of COVID-19 patients, similar to what was seen in MERS-CoV patients [35,104,105]. Coagulopathies and vascular disorders have been associated with hemorrhage in COVID-19 patients (Figure 2) [106]. It is also possible that reduced levels of ACE2 on endothelial cells of the brain microvasculature lead to blood coagulation and increased blood pressure, which may result in the rupture of blood vessels and hemorrhage (Figure 2) [97].

The increased risk of hypercoagulable states has resulted in the suggested addendums for COVID-19 patients at risk of cerebral vascular incidents. [107,108,109,110]. The documented endothelial injury, changes in circulating prothrombotic factors, and increased stasis resulting from immobilization due to COVID-19 infection have warranted hypervigilance in the monitoring and prophylactic treatment of these patients. The International Society on Thrombosis and Hemostasis, American Society of Chest Physicians, and American College of Cardiology have approved interim guidelines for prophylactic treatment and management. However, it is important to note these are interim guidelines until quality evidence, supporting interventions different from current standard practice, are identified [111,112,113].

Current recommendations for monitoring hospitalized at-risk patients include baseline complete blood count, levels of fibrinogen, D-dimer, prothrombin time, activated partial thromboplastin time, and inflammatory markers such as CRP and IL-6. The frequency of these tests is determined by the severity of the patient’s clinical presentations [114]. It is recommended that any abnormal findings in these measures are used for their prognostic value, and any changes to therapy should be the result of changes in signs or symptoms associated with stroke. Current treatment and management of patients presenting with active ischemic or hemorrhagic stroke do not differ from current recommendations, based on patients’ pre-existing conditions.

The implementation of prophylactic anticoagulant treatment varies depending on pre-existing conditions. The use of anticoagulants presents with its own adverse effects. The outpatient recommendations are not different from current guidelines (https://www.isth.org/page/Published_Guidance, accessed on 14 April 2021). However, as inpatient recommendations for the treatment and management of ischemic or hemorrhagic stroke due to COVID-19 are being evaluated, there is a consensus that thromboprophylaxis should be considered for all COVID-19 patients in intensive care units (ICUs) due to the increased risk of stasis [79,115].

### 5.2. Encephalitis, Acute Disseminated Encephalomyelitis, Encephalopathy, and Acute Necrotizing Encephalopathy

Encephalitis and meningitis are characterized by inflammation of the brain parenchyma and meninges, respectively [116]. The patient presents with headache, fever, vomiting, convulsions, and impaired sensations [60]. SARS-CoV-2 was detected in brain tissues and the CSF of COVID-19 patients who presented with meningitis or encephalitis, which indicates that the virus itself may cause this complication by infecting and damaging the brain (Figure 2) [40,41,42,60,117,118]. However, COVID-19 patients could also present with acute meningoencephalitis with no detectable SARS-CoV-2 or any other virus in the CSF [55,56,119]. The previous findings indicate that other mechanisms such as severe inflammation could be involved in the development of meningoencephalitis in COVID-19 patients. Based on the fatal consequences of encephalitis and meningoencephalitis, it should be considered as a possible complication in the management of COVID-19 patients. The early detection and treatment of meningoencephalitis are critical to prevent hemorrhagic encephalopathy that could be fatal.

Acute disseminated encephalomyelitis (ADEM) is another complication characterized by demyelination of CNS following viral infections particularly in children; however, occurrence in adults is reported [120]. MRI images of a 51-year-old woman, who has been diagnosed with COVID-19, showed several demyelinating lesions that are consistent with ADEM [121]. Post-COVID-19-ADEM was further confirmed by the CNS axonal damage and the lesions, in an autopsy of a 71-year-old COVID-19 patient, which are typical of ADEM [122].

Encephalopathy has also been described in 50% of hospitalized COVID-19 patients [123,124]. A study of several patients who died of COVID-19 showed that a significant number experienced hypoxic encephalopathy (Figure 2) [123]. Encephalopathy is more common in COVID-19 patients with coexisting or previous systemic and/or neurological complications [35,125]. Several cases that presented with altered mental state and confusion subsequent to COVID-19 did not have any evidence of CNS infection, which is typical of most cases of encephalopathy [125,126].

Acute necrotizing encephalopathy (ANE) often presents as neurological symptoms following viral infection, toxemia, and hypoxia [60]. Because SARS-CoV-2 infection results in viremia and hypoxia, it is not surprising that SARS-CoV-2 is a causative agent of encephalopathy (Figure 2) [60,97]. ANE was reported in cases with COVID-19, and pre-existing conditions could increase the risk of ANE [125]. A brain MRI of patients showed bilateral hemorrhagic rim-enhancing lesions in the thalamic temporal lobes and subinsular regions [127,128,129]. The cytokine storm that is associated with SARS-CoV-2 infection is believed to damage the BBB and cause brain necrosis in patients with severe COVID-19 (Figure 2) [127,128,129].

Evaluation of the current literature does not indicate any changes or interim recommendations for COVID-19 patients that differ from the current recommended guidelines for the treatment and management of encephalopathy. However, because encephalopathy has been identified as a frequent finding among older COVID-19 patients [130] and is associated with poorer outcomes among this cohort [3,131], there has been hypervigilance in testing for COVID-19 among these patients. Following the diagnosis of encephalitis, meningoencephalitis, or ANE, recommendations are to start with CSF PCR analysis for the presence of SARS-CoV-2 or other potential contributing viral infections such as Herpes Simplex Virus (HSV) [132]. Furthermore, the combined use of MRI and EEG appears to be very important in the detection of these cases [132].

### 5.3. Seizures

It is expected that some COVID-19 patients will develop seizures as a consequence of hypoxia, metabolic derangements, severe inflammation, organ failure, and cerebral affection (Figure 2) [41,133]. Indeed, seizures in COVID-19 patients have been reported due to SARS-CoV-2-induced brain damage, high levels of inflammatory mediators, and viral-induced encephalitis or meningitis [41,134,135,136]. Infection with SARS-CoV-2 reduces the seizures threshold which can worsen the case in epileptic patients or it can lead to seizures in patients with no history of seizures [137,138,139,140]. It is of note that seizures could be one of the initial symptoms in COVID-19 patients [141]. Focal seizures have been described in COVID-19 patients in addition to generalized tonic-clonic seizures [134]. Other than its presentation in adult COVID-19 patients, there were cases of seizures in COVID-19 children who present with fever or no fever (afebrile seizures) [142,143]. Therefore, it is important to consider seizures in the diagnosis of COVID-19 in children regardless of the presence or absence of fever [142,143]. The management of seizures could include the use of antiepileptic drugs and monitoring of seizures by electroencephalography especially in severe COVID-19 patients [135]. It is critical to diagnose and recognize the typical and atypical presentation of seizures in COVID-19 patients to better diagnose, treat, and avoid any long-term complications of seizures [144].

Current recommendations for the treatment and management of seizures and epilepsy for patients infected with COVID-19 do not differ from current guidelines. However, awareness of drug–drug interactions with COVID-19 treatment and the treatment for new or existing seizures must be considered when treating this patient population. The following discussion excludes the pediatric population because of limited early data that are reported among this group.

Many of the medications currently used in the treatment and management of COVID-19 induce, or inhibit, and are metabolized by the hepatic cytochrome P450 enzymes (CYP450). These enzymes are also altered or involved in the metabolism of many of the antiepileptic drugs (AEDs) frequently used in the treatment of seizure disorders.

Lopinavir/ritonavir are protease inhibitors used in the treatment of COVID-19 [145]. These drugs are frequently used in combination and have been shown to induce multiple CYP450 enzymes (CYP2C9, 2C19, 1A2, and 2B6) and glucuronyl transferase [146]. This activity decreases the plasma concentration of lamotrigine (via glucuronyl transferase) and possibly phenytoin and valproate (via CYP enzymes), which are frequently used AEDs [147,148]. Additionally, lopinavir/ritonavir plasma concentration may be reduced when used concomitantly with carbamazepine, phenytoin, and topiramate due to the ability of these AEDs to induce the CYP3A4 enzyme which metabolizes lopinavir/ritonavir [148].

Remdesivir is an adenosine analog that targets the RNA-dependent RNA polymerase and blocks viral RNA synthesis [145]. To date, there is limited information regarding the metabolism of remdesivir; however, it is partially metabolized via CYP3A4 (10%) [149]. This activity would result in reduced efficacy if used in combination with AEDs that induce this enzyme. Although there have been no drug interaction trials of remdesivir and concomitant AEDs it is important to note caution when used in combination with AEDs [150].

Currently, there is neither experimental nor clinical evidence for any noticeable drug interactions between AEDs and antivirals such as favipiravir, nitazoxanide, and interferon-beta which suggests that these antivirals do not require additional dosing considerations when used with AEDs in the management of COVID-19 patients presenting with seizures.

### 5.4. Altered Mental State (AMS)

Patients could present with confusion and delirium as early signs of COVID-19 without any of the respiratory symptoms [151]. Accordingly, the early detection of AMS may help in the proper treatment and prevention of COVID-19 spread. It has been estimated that 9% of COVID-19 patients have AMS [152]. We believe that AMS could be the result of direct invasion of the brain or damage resulting from high levels of inflammatory mediators due to the immune response to SARS-CoV-2 infection. We also believe that individuals with Alzheimer’s disease (AD) and related dementias are at high risk of COVID-19 and its associated morbidity and mortality. That could be attributed to the difficulty in applying disease prevention measures such as washing hands, social distancing, and isolation at home.

Helms et al. reported that 118 (84.3%) of 140 COVID-19 patients, who were treated in two intensive care units (ICUs) in France, had mental changes including delirium, agitation, and corticospinal tract signs [153]. MRI showed bilateral frontotemporal hypoperfusion. About 33% of the 45 survivors experienced a dysexecutive syndrome suggestive of the involvement of the frontal lobe, which is responsible for an individual’s mental state [153]. Based on the above findings, we believe that changes in mental status could be an important diagnostic for COVID-19 because COVID-19 patients may only present with delirium and confusion.

Patients with pre-existing or developing mental illness due to COVID-19 are expected to be treated with psychotropic drugs along with the standard treatment for the viral illness. Benzodiazepines (oxazepam and lorazepam), antidepressants (citalopram and escitalopram), antipsychotics (olanzapine), and the mood stabilizer (valproate) are suggested as safe considering the tolerability and minimal drug–drug interactions [154,155,156].

### 5.5. Guillain–Barré Syndrome (GBS)

GBS can occur following infections such as *Campylobacter jejuni*, Epstein–Barr virus, and *cytomegalovirus* due to molecular mimicry between peripheral nerve antigens and antigens of these pathogens [157]. Antipathogen antibodies can then cross-react with peripheral nerve antigens, causing inflammation and neuronal damage [157]. GBS has been described in several cases of COVID-19 patients, which manifest as weakness in the lower limbs and paresthesia and may progress to tetraparesis [49,158]. Nerve roots are typically involved, which is characterized by increased protein concentration in CSF and normal white blood cell count (cytoalbuminologic dissociation) [159,160]. It is of note that demyelinating polyradiculoneuropathy and/or axonal damage are characteristics of GBS in COVID-19 patients [161,162]. GBS may manifest in individuals with COVID-19 even before the appearance of the typical flu-like symptoms [163]. Gupta et al. described the difference between GBS due to COVID-19 and other types of GBS [164]. COVID-19 GBS is more prevalent in the elderly and males, and COVID-19 GBS patients may experience fever, cough, dyspnea, ageusia, hyposmia 5–14 days before the paresthesia, lower limb weakness, and facial weakness [164]. Unfortunately, COVID-19 GBS has residual weakness, dysphagia, and extended ICU stay than other GBS types. Variants of GBS such as Miller Fisher syndrome and polyneuritis cranialis have also been reported in COVID-19 patients [4,163,165].

Management of GBS is best achieved by intravenous immunoglobulin (IVIG) treatment [166,167]. Lopinavir/ritonavir use in COVID-19 with peripheral neuropathies is controversial because one study showed that protease inhibitors may increase the risk of peripheral neuropathy in patients with HIV [168]. However, other studies have found that lopinavir/ritonavir does not increase the risk of distal sensory polyneuropathy in HIV patients [169].

### 5.6. Skeletal Muscle and Neuromuscular Junction Complications

Severe inflammation in critically ill COVID-19 patients could lead to neuromuscular junction dysfunction and myopathy [170,171]. The invasion of muscle cells, which express the ACE2 receptor, is also a possible mechanism [170,171]. On the other hand, the risk of COVID-19 infection increased with the use of immunosuppressive/immunomodulatory therapies in patients with autoimmune neuromuscular disorders [172,173,174].

We studied different reports to propose a protocol for the management of myasthenia gravis (MG) and Lambert–Eaton myasthenic syndrome (LEMS) during COVID-19 [175,176] and concluded the following.

The MG expert panel suggests that decisions to manage every patient should be individualized, patients should take more precautions with extraordinary measures, and MG patients on immunosuppressive therapy should continue taking the medications. Hydroxychloroquine should be avoided in COVID-19 patients with MS or LEMS as the drug is reported to worsen MG [177,178]. The delay in initiation of the B-cell depleting therapy (rituximab) increases the risk of worsening myasthenia or myasthenia crisis [179,180].

### 5.7. Neurodegenerative and Demyelinating Disorders

It remains unclear whether SARS-CoV-2 infection is associated with the development of neurodegenerative diseases, such as multiple sclerosis (MS), Alzheimer’s disease (AD), and Parkinson’s disease (PD) [181]. There is also no evidence of the acceleration of these diseases in COVID-19 patients [181]. However, the high expression of ACE2 in CNS and the brain damage that SARS-CoV-2 causes could lead to long-term neurodegenerative diseases/complications [182]. MS is characterized by nerve demyelination and brain neurodegeneration due to immune-mediated inflammation [181]. The SARS-CoV-2-mediated neurological damage that results from inflammation or direct invasion of the brain is similar to that caused by MS [183,184]. However, there is not enough evidence that SARS-CoV-2 leads to MS or that MS patients are more susceptible to COVID-19, its CNS involvement, or the reactivation of MS lesions due to SARS-CoV-2-mediated immune dysregulation [185,186,187]. A case of a 67-year-old woman who had MS and died of COVID-19 showed that SARS-CoV-2 did not infect neuronal or glial cells and infection did not result in disease exacerbation or reactivation of MS lesions [187]. The findings of the previous case are consistent with other studies, which showed that COVID-19 did not affect the course of autoimmune diseases [185,186].

AD is another neurodegenerative disease that is characterized by neuroinflammation and neuronal loss and has many risk factors, which include age [188]. Several studies have shown that AD development could correlate with infections including viral infections [188]. Because SARS-CoV-2 infects/damages the CNS and induces severe inflammatory responses, it is possible that the long-term effect on cognitive function could develop in COVID-19 survivors [189]. To date, there is not enough evidence that SARS-CoV-2 causes or increases the risk of developing AD, as long-term studies are needed to draw this correlation. However, the infection of glutamate-producing and GABA-producing neurons by SARS-CoV-2 infection is a possible mechanism by which AD could develop secondary to COVID-19 [182].

Similar to AD, PD patients suffer from cognitive and memory issues in addition to the impairment of motor function [190]. Although ACE2 is widely expressed in CNS and SARS-CoV-2 infects and damages several sites in the brain, there is no direct evidence that SARS-CoV-2 induces or increases the risk of PD development or that PD patients are at higher risk of contracting SARS-CoV-2 [191,192]. There is not any evidence too that PD worsens during the course of COVID-19 disease [192]. However, it is highly possible that SARS-CoV-2 could be linked to PD development or its acceleration once more studies are conducted and follow-up of COVID-19 survivors is done over the next few years.

### 5.8. Miscellaneous Complications

The most common neurological symptoms associated with COVID-19 are headache, dizziness, myalgia, fatigue, hyposmia, hypogeusia, and visual impairment. These symptoms are seen in 30 to 45.5% of patients [35,193,194].

Headache is one of the most common neurological symptoms in COVID-19 patients and could be the first symptom of COVID-19 in a few patients (Figure 2) [35,195,196,197]. It occurred in 6-25% of COVID-19 patients depending on the study, and the intensity is often described as moderate to severe [152,198,199,200,201,202,203,204]. It has been noticed that headache as a result of COVID-19 starts as moderate pain due to systemic spread of the virus, whereas after a few days, severe inflammation could lead to photophobia and neck stiffness [205].

Past medical histories of several COVID-19 patients indicate that headache has been a regular complaint [199]. However, there are other cases in which COVID-19 patients never had any headache in their medical history and only experienced headache after SARS-CoV-2 infection, which suggests that headache is a complication of COVID-19 [206]. It has been suggested that headache occurs in COVID-19 patients as a result of SARS-CoV-2 infection of the nasal cavity trigeminal nerve endings [198]. Furthermore, headache could be due to infection of endothelial cells of the vessels in the trigeminovascular system [198]. The high level of proinflammatory cytokines could also irritate the trigeminal nerve endings leading to headache [198]. In our opinion, in addition to all the above-described mechanisms, headache could also occur due to lack of sleep, isolation, and anxiety in COVID-19 patients. Despite being a common symptom in COVID-19, headache can be easily treated by analgesics.

Dizziness is also reported as one of the common neurological symptoms that presents in 8–9% of COVID-19 patients (Figure 2) [35,207,208]. It is even reported, in some COVID-19 cases, to be more commonly occurring than headache [35]. A COVID-19 case for a 53-year-old woman was described, and dizziness was reported as an initial symptom along with dry throat, while fever, cough, and headache were absent [209]. Antiviral and other drug treatments resulted in case improvement. Therefore, it is important to watch for dizziness as one of the neurological complications that may help in the diagnosis of COVID-19 even in absence of respiratory symptoms.

Myalgia and fatigue have been commonly reported in COVID-19 patients (Figure 2) [35,193,194,207,208]. Depending on the study, fatigue was a complaint in 26–51% of patients, whereas 3–64% of patients had myalgia [210]. It has been postulated that myalgia in COVID-19 patients is due to severe inflammation and high levels of proinflammatory cytokines [211]. However, muscle invasion by SARS-CoV-2 remains a possibility because muscles express the ACE2 receptor. Some patients showed fatigue, muscle soreness, and elevated muscle enzyme levels such as creatine kinase all of which may be related to systemic inflammation and muscle damage [212].

Hyposmia (anosmia) and hypogeusia (ageusia) are loss of smell and taste, respectively, and they are among the most common early symptoms of COVID-19 (Figure 2) [213,214,215]. They are reported by up to 88% of COVID-19 patients with mild or moderate disease and therefore could be used for the diagnosis of COVID-19 [35,216,217,218,219]. Anosmia could appear as an initial symptom and is not accompanied by nasal inflammation [220,221]. Using MRI, an abnormal appearance of the olfactory bulb has been described in COVID-19 patients [220,221]. Infection of the olfactory epithelium and trigeminal nerves by SARS-CoV-2 may explain the loss of smell and taste in COVID-19 patients [222,223]. Because anosmia is highly prevalent and an early symptom of COVID-19, it can be used for the early diagnosis of COVID-19 [224]. This may help in the early isolation and treatment of COVID-19 patients, which could eventually result in a decline in the number of new cases.

Post-COVID-19 Neurological Syndrome (PCNS) indicates prolonged post-COVID-19 neurological symptoms. Several reports have shown that PCNS could present in the form of long-term symptoms that persist for months such as muscle pain and weakness, myopathy, sleep impairment, anxiety, depression, severe post-traumatic stress disorder (PTSD), dizziness, headaches, and anosmia [225,226]. The previous findings suggest that COVID-19 patients should be followed up after recovery for possible long-term post-COVID-19 neurological complications.

## 6. Mechanisms of SARS-CoV-2-Induced Neurological Complications

The high expression of ACE2 in the brain and peripheral nerves allows SARS-CoV-2 to infect the nervous system and cause neurological damage, which is manifested as complications secondary to SARS-CoV-2 infection [57]. Because ACE2 has several physiological functions, including the regulation of blood pressure, its usage by SARS-CoV-2 as an entry receptor may lead to its depletion and the accumulation of Ang II [227,228,229]. Elevated Ang II would result in increased blood pressure due to vasoconstriction and fluid retention [229]. Moreover, high levels of Ang II would promote inflammation and blood coagulation. Complications due to ACE2 depletion could be manifested as cerebrovascular diseases in COVID-19 patients (Figure 2).

Acute respiratory distress syndrome (ARDS), which occurs as a consequence of severe SARS-CoV-2 infection, could lead to hypoxia that can have deleterious effects on the brain, including edema, congestion, and neuronal degeneration (Figure 2) [64,202,230]. This hypoxia-induced brain damage is typically seen in hypoxic encephalopathy and ischemic stroke secondary to SARS-CoV-2 infection. However, it is important to note that direct damage of the brain by SARS-CoV-2 could also lead to respiratory failure and hypoxia [61]. One of the reasons behind ARDS is the severe inflammation due to the release of an excessive amount of proinflammatory cytokines that could be responsible for tissue damage in the lungs and other organs including the brain [202,231,232]. Severe inflammation was also noted locally in the brain after SARS-CoV-2 invasion due to the production of proinflammatory cytokines by astrocytes and microglia [21]. This also contributes to brain damage. Accordingly, therapies, such as IL-6 receptor monoclonal antibodies, which aim to reduce inflammation, have been used to prevent inflammation-dependent complications in COVID-19 patients [233].

Severe inflammation in COVID-19 patients, the infection of endothelial cells, and the activation of coagulation cascade could lead to hypercoagulability and disseminated intravascular coagulation (DIC) that is commonly seen in COVID-19 patients (Figure 2) [77,78,79,80,81]. The severe systemic inflammation on hospital admission could predict mortality in COVID-19 patients [87]. Stroke could be a consequence if anticoagulants are not administered [234,235]. A study demonstrated that serial systemic immune inflammation indices (SSIIi), which are determined based on neutrophil, platelet, and lymphocyte counts, are clinically correlated with PCNS events [236]. This implies that SSIIi could be used as a biomarker for many neurological complications including stroke.

Severe immunosuppression could be also implicated in COVID-19 patients with severe disease [237]. Circulating effector T cells were significantly reduced in COVID-19 patients [237]. In some patients, IL-6 was elevated but without elevations in other proinflammatory cytokines. It was noted too that blood mononuclear cells are less activated and produce lower levels of cytokines. All of the above suggests that immune responses may be impaired in some COVID-19 patients, which could lead to uncontrolled viral spread and tissue/organ damage including the CNS. Other studies reported an overproduction of proinflammatory cytokines in COVID-19 patients [238]. Moreover, another study found that in severe COVID-19 patients, there is a high level of anti-SARS-CoV-2 spike protein IgG antibodies [239]. This may indicate that antibody-dependent enhancement (ADE) of infection could play a role in mediating the infection of immune cells that express the Fcγ receptor for IgG [240,241]. Antibodies against SARS-CoV-2 can also cross-react with antigens in the nervous system causing complications such as GBS [158]. Based on these findings, overactivation of the immune system leads to hyperinflammation, whereas immunosuppression could result in the dissemination of SARS-CoV-2. Both of these mechanisms would eventually cause tissue damage.

## 7. Conclusions

The health care system is posed with a huge challenge of the current COVID-19 pandemic. Several neurological manifestations have been described in COVID-19 patients; however, more research needs to be performed to understand the pathogenic mechanism behind each of these disorders to better treat such patients with suitable drugs and in a timely manner. We believe that some of the available treatment options might potentially lead to a wave of neurological sequelae. Therefore, treatment of COVID-19 patients should consider the existing or the unknown neurological complications that may develop.
